# Disruption of Calcium Homeostasis in Cardiomyocytes Underlies Cardiac Structural and Functional Changes in Severe Sepsis

**DOI:** 10.1371/journal.pone.0068809

**Published:** 2013-07-23

**Authors:** Mara R. N. Celes, Lygia M. Malvestio, Sylvia O. Suadicani, Cibele M. Prado, Maria J. Figueiredo, Erica C. Campos, Ana C. S. Freitas, David C. Spray, Herbert B. Tanowitz, João S. da Silva, Marcos A. Rossi

**Affiliations:** 1 Departments of Pathology, Faculty of Medicine of Ribeirão Preto, University of São Paulo, São Paulo, Brazil; 2 Departments of Immunology and Biochemistry, Faculty of Medicine of Ribeirão Preto, University of São Paulo, São Paulo, Brazil; 3 Institute of Tropical Pathology and Public Health, Federal University of Goias, Goias, Brazil; 4 Departments of Urology, Albert Einstein College of Medicine, Yeshiva University, Bronx, New York, United States of America; 5 Department of Medicine (Cardiology), Albert Einstein College of Medicine, Yeshiva University, Bronx, New York, United States of America; 6 Department of Pathology, Albert Einstein College of Medicine, Yeshiva University, Bronx, New York, United States of America; UAE University, Faculty of Medicine & Health Sciences, United Arab Emirates

## Abstract

Sepsis, a major cause of morbidity/mortality in intensive care units worldwide, is commonly associated with cardiac dysfunction, which worsens the prognosis dramatically for patients. Although in recent years the concept of septic cardiomyopathy has evolved, the importance of myocardial structural alterations in sepsis has not been fully explored. This study offers novel and mechanistic data to clarify subcellular events that occur in the pathogenesis of septic cardiomyopathy and myocardial dysfunction in severe sepsis. Cultured neonatal mice cardiomyocytes subjected to serum obtained from mice with severe sepsis presented striking increment of [Ca^2+^]_i_ and calpain-1 levels associated with decreased expression of dystrophin and disruption and derangement of F-actin filaments and cytoplasmic bleb formation. Severe sepsis induced in mice led to an increased expression of calpain-1 in cardiomyocytes. Moreover, decreased myocardial amounts of dystrophin, sarcomeric actin, and myosin heavy chain were observed in septic hearts associated with depressed cardiac contractile dysfunction and a very low survival rate. Actin and myosin from the sarcomere are first disassembled by calpain and then ubiquitinated and degraded by proteasome or sequestered inside specialized vacuoles called autophagosomes, delivered to the lysosome for degradation forming autophagolysosomes. Verapamil and dantrolene prevented the increase of calpain-1 levels and preserved dystrophin, actin, and myosin loss/reduction as well cardiac contractile dysfunction associated with strikingly improved survival rate. These abnormal parameters emerge as therapeutic targets, which modulation may provide beneficial effects on future vascular outcomes and mortality in sepsis. Further studies are needed to shed light on this mechanism, mainly regarding specific calpain inhibitors.

## Introduction

Sepsis, a major cause of morbidity and mortality in intensive care units worldwide, is commonly associated with cardiac dysfunction, which worsens the prognosis dramatically for patients [1]. Hemodynamic changes, microcirculatory disturbances, systemic inflammatory cytokines, mitochondrial dysfunction, autonomic deregulation have been proposed as responsible for the depressed cardiac function in sepsis [2], [3]. However, conceptualizing cardiac depression in sepsis as simply the result of biochemical/functional changes oversimplifies the issue. Although in recent years the concept of septic cardiomyopathy has evolved [4]–[8], in which phenotypic changes develop in response to a variety of agents acting on myocardial fibers, the importance of myocardial structural alterations in sepsis has been largely ignored. Septic cardiomyopathy, unlike the classic cardiomyopathy, involves both left and right ventricles and is potentially reversible exhibiting depressed left ventricular (LV) systolic function and normal or low ventricular filling pressures [8] that depend on right ventricular dysfunction caused by acute lung damage and/or increased LV compliance.

A previous study from our laboratory detected sarcolemmal damage with increased permeability, as an early event in myocardial injury in cecal ligation and puncture (CLP)-induced severe sepsis in mice, due to oxidative damage to lipids and proteins [9], which could precede phenotypic changes characteristic of a septic inflammatory cardiomyopathy in man [5]. Afterwards, disruption of sarcolemmal dystrophin, the main component of the dystrophin-glycoprotein complex (DGC), associated with increased sarcolemmal permeability was demonstrated in hearts of septic mice [10]. The DGC confers structural stability to the myofiber sarcolemma and transmits force between sarcomeres and cell membrane to the extracellular matrix [11]. Possibly, dystrophin disruption aggravates sarcolemmal permeability making the sarcolemma more fragile and susceptible to damage, worsening the cell membrane disruption caused by oxidative damage in septic hearts, as part of a downward spiral. This idea is reinforced by the observation that sarcolemmal permeability improved in association with marked attenuation of dystrophin expression reduction in CLP septic mice treated with sodium dismutase, a superoxide scavenger [10].

Calcium homeostasis is essential to normal myocardium contraction/relaxation cycle. During myocyte contraction, Ca^2+^ enters the cell through L-type Ca^2+^ channels (I_Ca-L_) that trigger Ca^2+^-induced Ca^2+^ release (CICR) from the sarcoplasmic reticulum via activation of the cardiac ryanodine calcium-sensitive receptor [12]. As a consequence there is a rapid increase of intracellular free calcium ion concentration ([Ca^2+^]_i_) that promotes myofilament activation and myocyte shortening. However, disruption of homeostasis can cause cytosolic calcium overload due to leakage from the sarcoplasmic reticulum and/or increased cell membrane influx, which is toxic to cells and can cause cell death by activating intracellular calcium-dependent proteins, such as calpain, which degrades intracellular proteins, cellular membranes, and nuclear DNA [13], [14]. Disordered calcium homeostasis has been observed in *in vitro* studies in cardiomyocytes isolated from rat hearts 48 hours after CLP-induced sepsis [15], in cultured human adipocytes after lipopolysaccharide (LPS) stimulation [16], in *ex vivo* studies in smooth muscle cells of rat thoracic aorta after CLP-induced sepsis [17] and in sarcoplasmic reticulum from ventricles of rats treated with LPS [18], and in rat brains after peritoneal polymicrobial sepsis [19].

The present *in vitro* and *in vivo* studies were undertaken to test the hypothesis that alterations in calcium homeostasis in cardiomyocytes underlie cardiac structural and functional changes in severe sepsis. *In vitro* studies demonstrated changes in [Ca^2+^]_i_ and expression of calpain-1 and dystrophin in neonatal cultured cardiomyocytes after adding sera from septic compared to sham-operated mice to the cultures. *In vivo* studies using the calcium channel blocking drugs verapamil (VP) to selectively inhibit the influx of calcium in the myocardial cells [20] and dantrolene (DT) to abolish excitation-contraction coupling in muscle cells by inhibiting calcium release from sarcoplasmic reticulum by blocking the cardiac type-2 ryanodine receptors (RyR2) [21] restored or prevented altered expression of calpain-1, dystrophin, and the contractile proteins sarcomeric actin and myosin in response to septis. Moreover, VP and DT prevented the compromised cardiac function and extended survival of mice submitted to severe sepsis induced by CLP compared to sham-operated controls.

## Materials and Methods

### Ethics statement

The animal protocols were approved by the Committee on Animal Research of the Faculty of Medicine of Ribeirão Preto, University of São Paulo, Brazil (Protocol#s 120/2009 and 183/2010) and by the Institutional Animal Care and Use Committee of the Albert Einstein College of Medicine, New York, USA and complied with the Guide for the Care and Use of Laboratory Animals published by the National Institute of Health (NIH Publication No 85-23, revised 1996). All efforts were made to minimize animal suffering.

### Mice

Male C57BL/6 mice weighing 22–25 g were maintained at a constant temperature (22±2°C) and a 12:12h light-dark cycle. They were housed at the animal Facility of the Department of Pathology of the Faculty of Medicine of Ribeirão Preto or at the Institute for Animal Studies of the Albert Einstein College of Medicine and given standard mouse chow and water *ad libitum*. Primary culture of neonatal mouse cardiomyocytes was performed with cardiomyocytes obtained from neonatal C57BL/6 mice from the breeding colonies of the Faculty of Medicine of Ribeirão Preto and of Einstein College of Medicine.

### Polymicrobial sepsis (CLP model) and blood collection

A modified CLP model was used to induce polymicrobial sepsis [22]. Briefly, mice were anesthetized with 2.0% isoflurane vaporized in medical oxygen (O_2_) via facemask. The abdomen was shaved and a midline incision performed. The cecum was isolated and ligated with 6–0 silk thread below the ileocecal valve without causing bowel obstruction and punctured with an 18-gauge needle to induce severe septic injury (SSI). Bowel content was gently extruded through the puncture and the cecum was then replaced to its original position and the abdomen sutured. Sham-operated animals (controls) underwent the same procedures, except for cecal ligation and puncture. All mice received subcutaneous doses of saline (50 mL/kg of body weight) immediately and 12 hours after surgical procedure to prevent dehydration. Sodium dipirone solution (10 mg/100 g body weight, i.p.) was administered at the start of surgery, then 6 and 14 hours post-surgery for pain relief. Rats were awakened from anesthesia, monitored to ensure adequate recovery, and returned to the animal facility. Mice were monitored daily for signs of disease, which typically included piloerection, hunched gait, lethargy and eye discharge. Mice displaying severe signs of distress (labored breathing, non-responsiveness to cage tapping, failure of grooming and severe eye discharge) were humanely sacrificed by injecting a mixture of ketamine (90–120 mg/kg) and xylazine (10 mg/kg) followed by cervical dislocation. The survival rate was monitored each 12 hours for 5 days after surgery using 8 animals per group. Six hours after CLP, vein tail blood samples from septic and sham-operated mice were collected, centrifuged at 3000 rpm at 4°C for 5 min, and the serum stored at −80°C.

### *In vitro* experiments

#### Primary culture of neonatal mouse cardiomyocytes and treatments

Cardiomyocytes were isolated from neonatal mice hearts as described previously [23]. Briefly, 20 neonatal mice were killed by decapitation, the hearts isolated and minced in the dissociation solution containing 1.25% pancreatin (GIBCO, Grand Island, NY, USA) and 300 mg/L bovine serum albumin (BSA; Sigma-Aldrich, Inc., St. Louis, MO, USA) diluted in (in g/L) 8.0 NaCl, 0.2 KCl, 0.05 Na_2_HPO_4_, 1.0 NaHCO_3_, and 2.0 dextrose. The homogenate was transferred to an Erlenmeyer flask with the dissociation solution and placed in a water bath while continuously stirring. The supernatant fraction from each digestion period was spun at 1200 rpm for 10 min, and the pellet re-suspended in Dulbecco's modified Eagle's medium (DMEM) containing 10% fetal bovine serum (FBS) (GIBCO) and 1% penicillin/streptomycin (GIBCO). This procedure was repeated 5–7 times and then the tube with dissociated cells was placed in the incubator (37°C, 5% CO_2_). The cells were pooled and plated in 100 mm culture dishes for 2 hours. Unattached 5×10^5^ cells/cm^3^ or 5×10^4^ cells/cm^3^ highly rich in myocytes were then collected from the 100 mm dishes, plated in 6-well cell culture plates (Corning, NY, USA) and in glass bottomed dishes (MatTek, Ashland, MA USA) or in Permanox Lab-Teks chamber slides (Nalgene Nunc International, Rochester, NY, USA), respectively, and placed in the incubator for 24 hours. After this period, the medium was replaced by DMEM supplemented with cytosine b-D-arabino-furanoside (Sigma-Aldrich). After 48 hours, 85–90% of plated cardiomyocytes showed spontaneous contractility. After 5 days the culture medium was removed and new serum-free medium supplemented with 10% (v/v) serum from either septic or sham-operated mice was immediately added and left in the incubator for additional 2 minutes or 24 hours. Following the experimental periods, the cardiac cells were washed and processed for Western blotting, immunofluorescence labeling for detection of calpain-1 and dystrophin, and measurement of [Ca^2+^]_i_ in response to septic serum.

#### Imaging of intracellular calcium transients

The [Ca^2+^]_i_ in cardiomyocytes was measured as described previously [24], [25]. Briefly, neonatal cardiomyocytes cultured on glass bottom dishes (MatTek) were loaded with the ratiometric calcium indicator Fura-2-acetoxymethyl ester (Fura-2 AM, 10 μM, Molecular Probes, Eugene, OR) in DMEM without phenol red (Invitrogen-GIBCO) for 45 min at 37°C in a CO_2_ humidified incubator. The cultures were then rinsed three times with Tyrode solution [(in mM) 137.0 NaCl, 2.7 KCl, 0.5 MgCl_2_, 1.8 CaCl_2_, 12.0 NaHCO_3_, 0.5 NaH_2_PO_4_, 5.5 glucose, and 5 HEPES; pH 7.1–7.2] to remove the unincorporated indicator and kept in Tyrode solution throughout the experiments. Dishes were then placed on the stage of a Nikon TE2000-U inverted microscope (Japan) equipped with a 20× S Fluor objective (Nikon, Japan), Fura-2 filter set (Chroma, Bellows Falls, VT, USA), CoolSnap HQ^2^ CCD camera (Photometrics, Tucson, AZ, USA) and shutter system (Lambda DG-4, Sutter Instruments, Novato, CA, USA) driven by a computer through Metafluor software (Molecular Devices, Downingtown, PA, USA). Changes in Fura-2 fluorescence intensities emitted at two excitation wavelengths (340 and 380 nm) were acquired at 1.0 Hz and [Ca^2+^]_i_ was determined from Fura-2 ratio images using an *in vitro* calibration curve as previously described [25]. Cardiomyocytes were continuously imaged for 5 min before and 5 min after the cells were stimulated with either 10% serum from septic mice or 10% serum from sham-operated mice diluted in Tyrode solution. Effects of prolonged, 24 hour exposure to 10% serum from septic or control mice on cardiomyocyte intracellular calcium transients were also investigated. For this, serum was added to the cultures 24 hours prior to imaging and maintained throughout the loading of cells with Fura-2 AM, washing of the calcium indicator and imaging steps. Cells were then imaged for 5 minutes without interruption, as described above. Effects of serum on cardiomyocytes was evaluated as changes in basal [Ca^2+^]_i_ levels divided by the number of transients imaged in the 5 min duration of the recordings.

#### Immunofluorescence

Neonatal cardiomyocytes seeded in Lab-Tek chamber slides were stimulated with serum obtained from septic and sham-operated mice. After septic or sham serum challenge, cells were fixed with 4% paraformaldehyde for 20 min, and then washed three times with 4°C cold PBS. Cells were permeabilized with 0.5% Triton X-100 (Sigma-Aldrich) and non-specific staining was blocked with 4% BSA. Primary antibodies to calpain-1 (goat polyclonal antibody anti-calpain-1, 1∶100; Santa Cruz Biotechnology, Santa Cruz, CA, USA) and dystrophin (rabbit polyclonal antibody anti-dystrophin, 1∶200; Santa Cruz Biotechnology) were incubated overnight at 4°C; following, the cells were washed and incubated with fluorescein conjugated secondary antibodies (anti-goat IgG, diluted 1∶500 or anti-rabbit IgG, diluted 1∶500; Vector Laboratories, Burlingame, CA, USA). F-actin filaments were stained with Alexa fluor 594 conjugated phalloidin (Invitrogen-GIBCO) for 1 hour at room temperature and DNA was stained with DAPI. The images were analyzed with Leica DM 6000 M microscope equipped with Leica AF6000 Deconvolution System (Leica Microsystems).

#### Western blotting

To measure calpain-1 and dystrophin protein expression levels in cultured cardiomyocytes 24 hours after serum stimulation, the cells (n = 3 distinct cardiomyocyte cultures/group) were removed from plates and lysed with protease inhibitor cocktail (Sigma-Aldrich) using cell lifter in extraction buffer. Total cell protein (20μg of protein/well) were resolved on a 5% or 10% SDS-Page gel and transferred to PVDF membrane (Amersham Pharmacia Biotech, Amersham, UK). The membranes were blocked with 5% albumin for 2 hours and incubated overnight at 4°C with the primary antibodies anti-calpain-1 (goat polyclonal antibody, 1∶500; Santa Cruz Biotechnology) and anti-dystrophin (rabbit polyclonal antibody, 1∶500; Santa Cruz Biotechnology) followed by incubation with HRP-conjugated secondary antibodies goat anti-rabbit IgG and rabbit anti-goat IgG (1∶10,000; Santa Cruz Biotechnology) for 1 hour at room temperature. To control for lane loading, the same membranes were probed with rabbit anti-tubulin (1∶1,000; Santa Cruz Biotechnology) or anti-goat anti-GAPDH (1∶10,000, Cell Signaling, Danvers, MA, USA) after being washed with stripping buffer. The membranes were developed using ECL (Millipore, Billerica, MA, USA). Gel documentation and signals quantification were made using the Bio-Image Analysis of Molecular Imager ChemiDoc XRS System (Bio-Rad, Richmond, CA, USA). The results were expressed as relative integrated intensity compared with that of control hearts measured in the same batch.

### *In vivo* experiments

#### Groups and drugs

Male C57BL/6 mice were allocated into six groups: 1) sham; 2) SSI; 3) sham+verapamil (SH+VP); 4) SSI+verapamil (SSI+VP); 5) sham+dantrolene (SH+DT) and 6) SSI+dantrolene (SSI+DT). Half the mice received intraperitoneal (i.p.) injection of either verapamil hydrochloride (5 mg/Kg body weight, Sigma-Aldrich Co. St. Louis, MO, USA) or dantrolene sodium salt (10 mg/Kg body weight, Sigma-Aldrich) diluted in sterile 0.9% NaCl saline (100 µl total volume/animal) two hours after CLP surgery or sham-operation. Control groups (sham and SSI) received an equivalent volume of saline alone.

#### Western blotting

To determine the amount of calpain-1, dystrophin, sarcomeric actin and cardiac myosin heavy chain (MHC) in the hearts of untreated and treated septic and sham-operated mice (n = 3–5 animals/group), Western blotting was performed 6 and 24 hours after CLP procedure or sham-operation. The animals were anesthetized with ip injection of ketamine (87.5 mg/kg) and xylazine (2.5 mg/kg), the hearts were collected and washed in cold saline and the left ventricles were isolated and homogenized in extraction buffer with protease inhibitor cocktail (Sigma-Aldrich).

Total protein (50 µg protein/well) was resolved on 5% or 10% SDS-Page gels and transferred to PVDF membrane (Amersham Pharmacia Biotech, Amersham, UK). The membranes were blocked with 5% albumin for 2 hours and incubated overnight at 4°C with the primary antibodies: anti-calpain-1 (goat polyclonal antibody, 1∶500; Santa Cruz Biotechnology), anti-dystrophin (rabbit polyclonal antibody, 1∶500; Santa Cruz Biotechnology), anti-α-sarcomeric actin (goat polyclonal antibody, 1∶1000; Sigma-Aldrich) and anti cardiac myosin heavy chain (MHC) (goat polyclonal antibody 1∶2000) (Sigma-Aldrich) followed by incubation with HRP-conjugated goat anti-rabbit IgG or rabbit anti-goat IgG secondary antibodies for 1 hour at room temperature. The loading control, documentation, and quantification of membranes were performed as described above.

#### Electron microscopy

Small blocks (1 mm^3^) of myocardial tissue from the left ventricular free wall of mice (n = 4 animals/group) 24 h after septic injury and sham-operation were fixed with 2.5% glutaraldehyde in cacodylate buffer and post-fixed in 1% osmium tetroxide. Ultrathin sections were double-stained with uranyl acetate and lead citrate and examined in a Zeiss EM109/900 electron microscope (Carl Zeiss, Oberköchen, Germany) at 80 kV.

#### Echocardiography

Transthoracic echocardiography was used to evaluate left ventricular (LV) function in untreated and treated septic and sham-operated mice (n = 7–10 animals/group), immediately before surgery and 12 and 24 hours after CLP procedure or sham-operation. Mice were lightly anesthetized with inhaled isoflurane (1.5%) vaporized in medical O_2_. Thoracic hair was removed with a topical depilatory agent and ultrasound gel was placed to form an interface between chest and a RMV 707B High-Frame-Rate Scanhead (frequency band 15–45 MHz) from Vevo 770 System or a MS 400 High-Frame-Rate transducer (frequency band 18–38 MHz) (Visual Sonics, Toronto, ON, Canada). Heart rate (HR) and body temperature (°C) were continuously monitored. The mice core temperatures were maintained normothermic using a heat-controlled platform. High-resolution one-dimensional M-mode images as well as high-resolution two-dimensional electrocardiogram (ECG) based kilohertz visualization (EKV) B-mode were acquired. High-resolution B-mode images were used to measure the area and volume of the LV and to calculate the ejection fraction (EF), fractional shortening (FS) and cardiac output (CO).

### Statistical analysis

All data are presented as mean ± SD. Multiple comparisons were made using a one-way analysis of variance (ANOVA) followed by Tukey or Bonferroni post-tests. For comparison of two groups Student’s *t*-test was used. The survival rates were constructed using the Kaplan-Meier curve, and differences in mortality were compared using the log-rank (Mantel-Cox) test. p<0.05 was considered statistically significant.

## Results

### Intracellular calcium concentration

**Figure 1:**
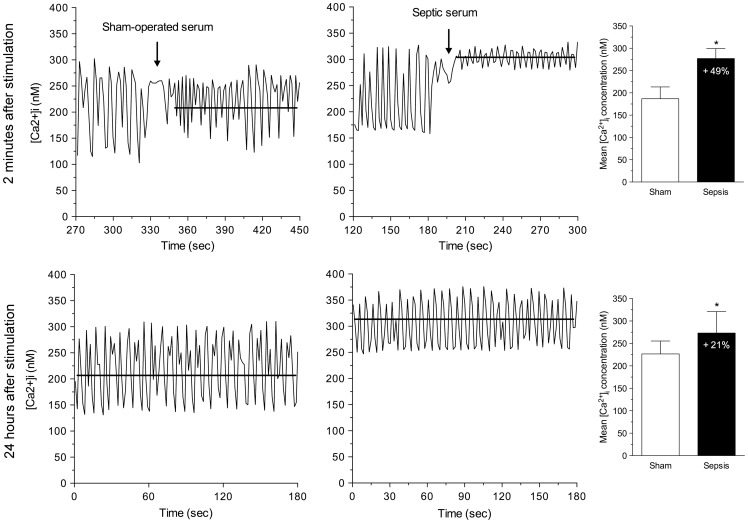
Increment of mean [Ca^2+^]_i_ after addition of septic serum from mice. Cardiomyocytes exposed to serum from septic mice for 2 minutes (upper panel) presented a marked increment of mean [Ca^2+^]_i_, around 49%, due to a rise of diastolic ion levels, with decreased amplitude of the calcium transients as compared to cardiomyocytes incubated with serum from sham-operated mice. Cardiomyocytes exposed to septic serum for 24 hours (bottom panel) demonstrated an effective mean increase of [Ca^2+^]_i_, around 21%, due to a rise of both systolic and diastolic ion levels, but the amplitude of systolic and diastolic [Ca^2+^]_i_ levels were similar in both experimental and control conditions (*, P<0.01).

Changes in [Ca^2+^]i were evaluated by epifluorescence microscopy using the ratiometric probe Fura-2-AM in cultured neonatal cardiomyocytes after addition of sera from both sham-operated and septic mice. Cardiomyocytes stimulated with serum from SSI mice exhibited a significant increase of mean [Ca^2+^]i of about 49% and 21% at 2 minutes and 24 hours, respectively, as compared to mean [Ca^2+^]_i_ in cultured cardiomyocytes subjected to serum from sham-operated mice. At 2 minutes after septic serum addition to the culture, a marked increment of mean [Ca^2+^]_i_ was observed due to increased basal (diastolic) levels of the ion, resulting in a strikingly lower amplitude of Ca^2+^ transients compared with that of control cultured cardiomyocytes, with no change in frequency of Ca^2+^ transient oscillations. In contrast, at 24 hours after septic serum addition, the cardiomyocytes showed higher mean basal Ca^2+^ levels but similar amplitudes and frequency of transient Ca^2+^ oscillations (Figure 1).

### *In vitro* Immunofluorescence and Western blotting

**Figure 2:**
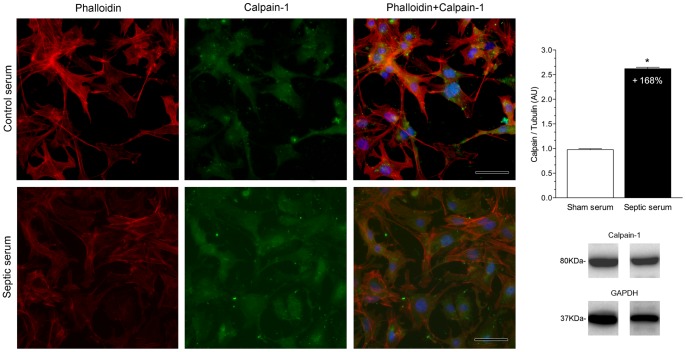
Increased expression and amount of calpain-1 in cultured cardiomyocytes stimulated with septic serum. The fluorescent signal for calpain-1 disclosed increased in cardiomyocytes challenged with serum from septic mice (bottom panel, green fluorescence) in comparison with the fluorescent signal in cardiomyocytes stimulated with serum from sham-operated mice (upper panel, green fluorescence). F-actin was stained with Alexa Fluor 594 dye (red fluorescence) and DNA with DAPI (blue fluorescence). Western blotting demonstrated a strikingly increased, around 168%, of calpain-1 amount in cardiomyocytes stimulated with serum from septic animals compared to cardiomyocytes stimulated with serum from sham-operated mice (*, P<0.01). GAPDH was used as a protein loading control. Data are expressed as mean ± S.D. Scale bars indicate 50 μm.

**Figure 3:**
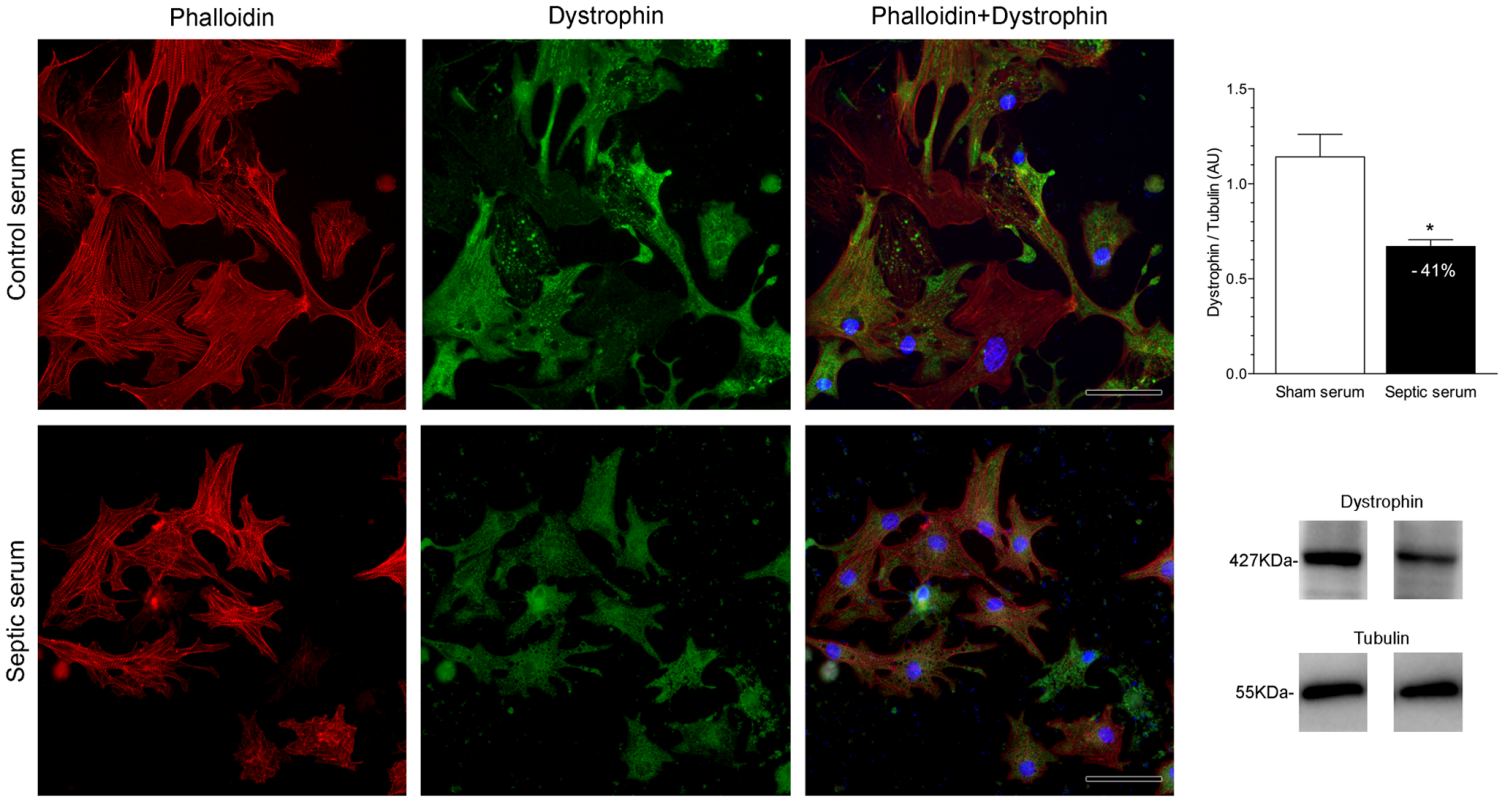
Decreased expression and amount of dystrophin in cultured cardiomyocytes stimulated with septic serum. The fluorescent signal for dystrophin (green fluorescence) presented decreased expression (bottom panel) with bleb formation in cardiomyocytes stimulated with septic serum in comparison with the fluorescence expression in cardiomyocytes expose to serum from sham-operated mice (upper panel). F-actin was stained with phalloidin conjugated Alexa Fluor 594 dye (red fluorescence) and DNA with DAPI (blue fluorescence). Alexa Fluor 594 staining showed disruption and rearrangement of F-actin filaments. Western blotting demonstrated that dystrophin amount was markedly decreased, around 41%, in cardiomyocytes stimulated with serum from septic mice compared to amounts observed in cardiomyocytes stimulated with serum from sham-operated mice (*, P<0.01). α-Tubulin was used as a protein loading control. Data are expressed as mean ± S.D. Scale bars indicate 50 μm.

Calpain-1 and dystrophin expression in neonatal cardiomyocytes were evaluated 24 hours after adding serum from septic or control mice [(10% (v/v)]. The immunofluorescence analysis clearly showed an increased expression of calpain-1 in cardiomyocytes submitted to septic serum challenge as compared with cardiomyocytes treated with control serum. Western blots showed a more than 2.5 fold increment in calpain-1 level in cultured cardiomyocytes subjected to septic serum in comparison to those subjected to control serum (figure 2), corroborating the immunofluorescence results. Immunostaining showed lower dystrophin expression in cultured neonatal mice cardiomyocytes exposed to septic serum compared to cardiomyocytes exposed to normal serum. As is particularly evident in the overlaid images, the loss/reduction of dystrophin expression was associated with cytoplasmic vacuolization and disruption of actin filaments. Western blotting revealed that dystrophin abundance was significantly lower (by about 40%) in cardiomyocytes stimulated with septic serum compared to cardiomyocytes stimulated with serum from sham-operated mice (figure 3).

### *In vivo* Western blotting

**Figure 4:**
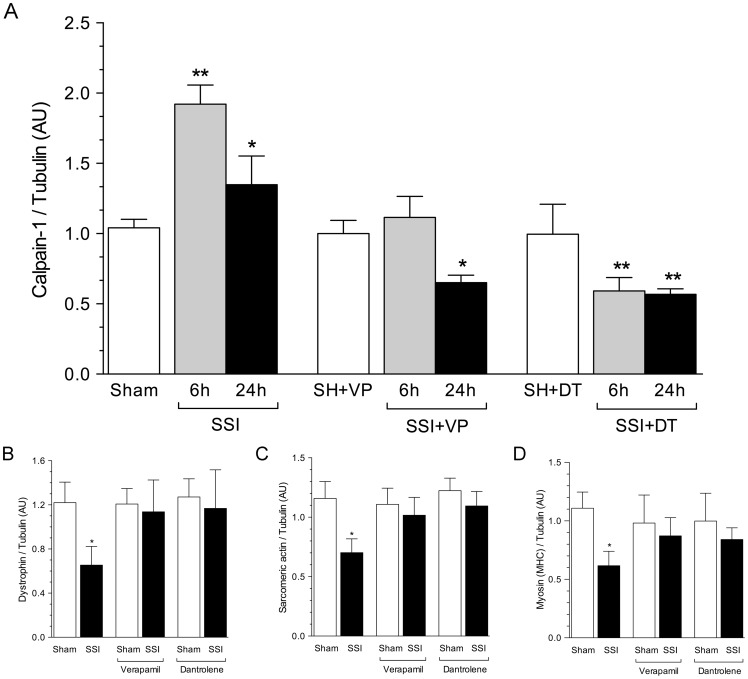
Verapamil (VP) and dantrolene (DT) abrogated calpain-1, dystrophin, sarcomeric actin, and myosin heavy chain changes induced by severe sepsis in mice. (The mean amount of calpain-1 in hearts from untreated mice (Sham) or in hearts of mice treated with either verapamil (SH+VP) or dantrolene (SH+DT) at 6 and 24 hours after sham operation were absolutely identical regarding each procedure and computed and represented as a single column). **A.** The mean amounts of calpain-1 in SSI hearts at 6 and 24 hours after CLP procedure were markedly increased in comparison with the mean amount in hearts of control mice after sham operation. In contrast, the mean amount of calpain-1 in SSI hearts from mice treated with verapamil at 6 hours after surgery was similar to the mean amount of calpain-1 in hearts from sham-operated treated with verapamil. The mean amount of calpain-1 in septic hearts from mice treated with verapamil at 24 hours after CLP procedure was significantly inferior to the mean amount of calpain-1 in hearts from sham controls. The mean amounts of calpain-1 in SSI hearts from mice treated with dantrolene at 6 and 24 hours after operation were significantly lower in comparison with the mean amount of calpain-1 in sham controls treated with dantrolene. **B.** The mean amount of dystrophin in SSI hearts was significantly reduced in comparison with the mean value in sham controls. The mean amounts of dystrophin in SSI hearts from mice treated with either verapamil or dantrolene were similar to those observed in sham-operated mice treated with either verapamil or dantrolene and sham-operated untreated mice. **C and D.** The mean amounts of actin and myosin in SSI hearts were markedly reduced compared to sham control values. Contrasted with these results, the values of sarcomeric actin and myosin in SSI hearts from mice treated with either verapamil or dantrolene were similar to those disclosed in hearts from sham-operated mice treated with verapamil or dantrolene and sham-operated untreated mice. (*, P<0.01; **, P<0.001).

The average amount of calpain-1 in mice heart ventricles, relative to tubulin was evaluated 6 and 24 hours after sham-operation or severe septic injury in untreated (sham SH) and treated (SSI) mice with either verapamil (VP) or dantrolene (DT). Mean calpain-1 amount was significantly more than 80% higher in SSI mice at 6 hours after CLP when compared with the values observed in sham control ventricles. Twenty-four hours after CLP the mean calpain-1 amount in SSI ventricles was increased by about 30% (Figure 4A). By contrast, calpain-1 expression in ventricles of SSI mice treated with verapamil (SSI+VP) at 6 hours after CLP was similar to that of sham-operated mice given verapamil and at 24 hrs after treatment, calpain-1 in SSI mice treated with verapamil was significantly reduced than in sham-operated treated mice. Calpain-1 levels in SSI mice treated with dantrolene (SSI+DT) at 6 and at 24 hours after CLP were significantly reduced in comparison with sham-operated mice treated with dantrolene. Dantrolene treatment also decreased calpain-1 at 6 and 24 hours after CLP compared to SSI group (Figure 4A).

Dystrophin, sarcomeric actin and myosin in mice heart ventricles, with respect to tubulin were evaluated 24 hours after sham-operation or CLP in untreated verapamil- and dantrolene-treated mice.

Dystrophin levels in SSI heart ventricles were reduced by about 50% when compared with that of sham-operated mice 24 hours after CLP. Treatment of SSI mice either with verapamil or dantrolene significantly prevented the loss of dystrophin in heart ventricles with no difference compared to ventricles of sham-operated and sham-treated with VP (1.20±0.14 AU) and DT (1.27±0.16 AU) (Figure 4).

Sarcomeric actin was about 30% lower in SSI mouse heart ventricles compared to that of sham-operated mice 24 hours after CLP. Verapamil or dantrolene treatment abrogated the reduction of sarcomeric actin seen in SSI hearts, with values similar to those in ventricles of sham-operated and sham-treated with VP and DT (Figure 4B).

In addition, myosin protein was reduced by about 40%, in SSI mice ventricles compared to those of sham-operated mice 24 hours after CLP. Treatment of septic mice with either verapamil or dantrolene prevented the reduction of MHC protein in heart ventricles seen in the SSI group, with values not different from those observed in sham-operated and sham-operated treated mice with VP (Figure 4C).

### Electron microscopy

Protein degradation within the heart occurs through three main pathways: (a) the calpain system, (b) the ubiquitin-proteasome system (UPS), and (c) autophagy/lysosomal degradation. These three systems degrade protein using distinct but complementary mechanisms. The process of autophagy degrades larger proteins such as sarcomere proteins and organelles that the UPS is unable to handle while calpain-mediated proteolysis is mediated by a family of calcium-dependent cysteine proteases present in all cells. Taking into account previous results from our laboratory [9], [10] and the present ones demonstrating degradation (proteolysis) of sarcomeric actin and myosin, the myocardium of mice 24 hours after severe sepsis was examined under the electron microscope in order to evaluate autophagy/lysosomal degradation as compared with sham-operated controls.

**Figure 5:**
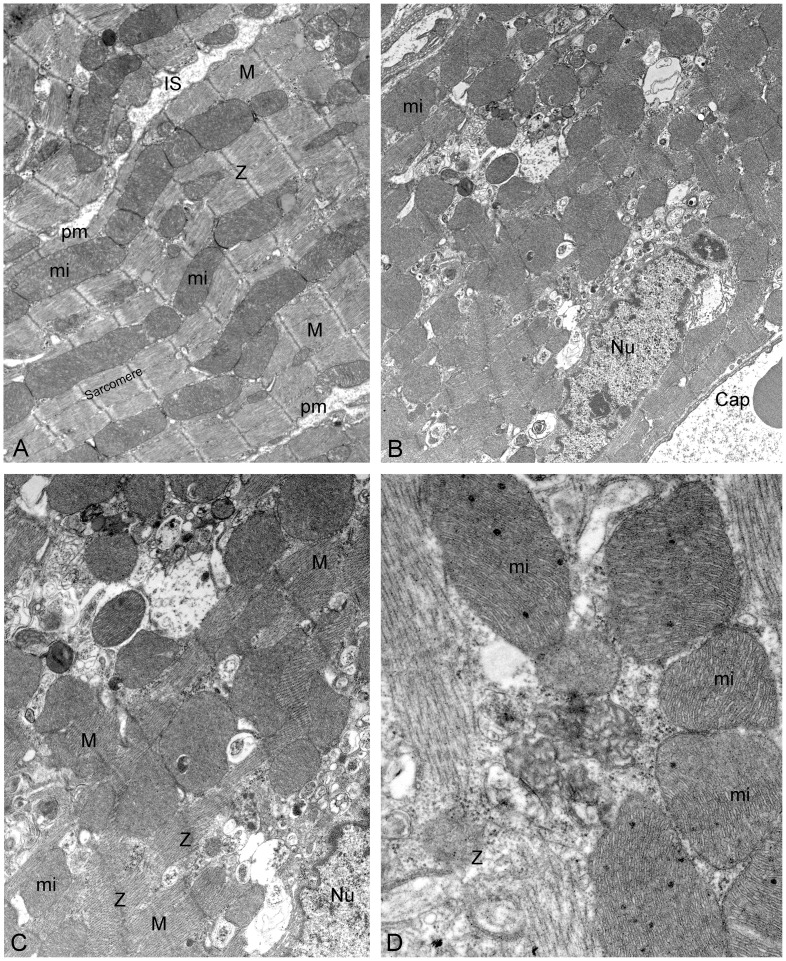
Electron microscopic study of the mice myocardium 24 hours after septic injury and sham-operation. **A.** The ultrastructural examination revealed that the myocardium from control mice did not differ from that reported in the literature presenting continuous and regularly spaced myofibrils that alternate with serially arranged, elongated mitochondria (mi). Sarcomeres are clearly delimited by two neighboring well-defined Z-bands. Interfibrillar mitochondria are closely related to the sarcomere. Magnification bar represents 2 µm. **B.** The ultrastructure of the myocardium of mice with severe sepsis 24 hours after CLP showed ill-defined sarcomeres with abundant accumulation of autophagolysosomes within cardiomyocytes (arrows). Magnification bar represents 2 µm. **C.** Higher magnification showing autophagolysosomes (arrows), foci of ill-defined and disorganized/disrupted sarcomeres and blurring/breakage of Z-bands (Z). Magnification bar represents 1 µm. **D.** Increased number of electron-opaque calcium granules within mitochondria (arrow heads), breakage of Z band (Z) and autophagolysosome (arrow). Magnification bar represents 500 nm. M, M line; Nu, nucleus; pm, plasma membrane; IS, interstitial space; Cap, capillary.

Electron microscopy of myocardium from control mice was similar to that reported in the literature [26]. However, the hearts from mice with severe sepsis 24 hours after CLP showed abundant accumulation of autophagolysosomes within cardiomyocytes, their number varying from cell to cell, with many myofibers without any inclusion and many others with a variable number of vacuoles spread out through the sarcoplasm of cardiomyocytes, most of them close to the nuclei. Moreover, the cardiomyocytes of septic hearts displayed diffuse foci of disorganized/disrupted sarcomeres, with blurring and breakage of Z-bands and increased number of electron-opaque calcium granules within mitochondria (figure 5).

### Cardiac function (ECHO)

Using two-dimensional and M-mode echocardiography, the ejection fraction-Teichholz (EF) and fractional shortening (FS) were calculated and used as a determinant of LV cardiac function immediately before and 12 and 24 hours after CLP surgery.

**Figure 6:**
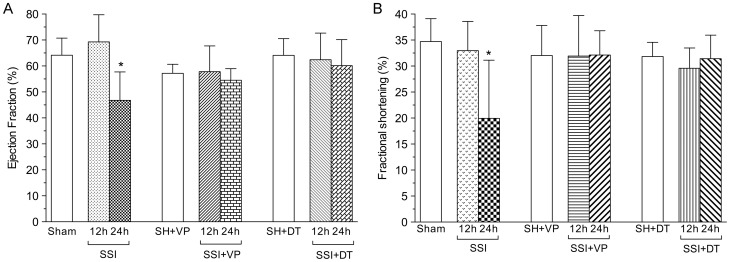
Verapamil and dantrolene restrained ejection fraction (EF) and fractional shortening (FS) changes induced by severe sepsis in mice. (The left ventricular ejection fraction and fractional shortening in untreated mice (Sham) or in mice treated with either verapamil (SH+VP) or dantrolene (SH+DT) at 12 and 24 hours after sham operation were absolutely identical regarding each procedure and computed and represented as a single column). **A and B.** The left ventricular ejection fraction and fractional shortening in mice submitted to CLP-induced sepsis were similar to the values in mice submitted to sham operation at 12 hours after surgical procedure. But the left ventricular ejection fraction and fractional shortening in SSI mice was markedly reduced in comparison with the mean values observed in sham controls at 24 hours after surgery. In contrast, the treatment with either verapamil or dantrolene abrogated the left ventricular ejection fraction and fractional shortening in SSI mice that presented mean values similar to those observed sham-operated mice treated with either verapamil or dantrolene and sham-operated untreated mice. (n = 7 to 10 in each group; *, P<0.01).

Figure 6 shows that at 12 hours after CLP, the mean ejection fraction (EF) from SSI mice (69.27±10.48%) was similar to the value found in sham-operated mice. At 24 hours after severe septic injury, the mean EF was significantly decreased by about 27% as compared to sham-operated controls. Septic mice treated with either verapamil or dantrolene showed statistically similar values of EF at 12 hours and at 24 hours after CLP. Moreover, these mean EF values of SSI treated mice were similar to the values observed in sham-operated controls and sham-operated mice treated with VP or DT (Figure 5). The mean values of EF of septic mice treated with either verapamil or dantrolene at 24 hours after CLP were significantly improved in comparison when compared to the untreated SSI group in the same period (Figure 6).

The mean fractional shortening (FS) values of SSI mice were not statistically different from values observed in sham-operated mice 12 hours after CLP surgery. At 24 hours after SSI injury, the mean FS was about 42% lower than the mean FS in sham-operated controls. Similar mean values of FS were observed in septic mice given either verapamil or dantrolene at 12 hours and 24 hours after CLP surgery. Additionally, these mean FS values were not different from those observed in sham-operated mice untreated or treated with VP or DT (Figure 6). The mean values of FS from septic mice treated with either verapamil or dantrolene at 24 hours after CLP were significantly improved in comparison with the SSI group in the same period (Figure 6).

Table 1 shows the mean cardiac output (CO), heart rate (HR) and body temperature (°C) evaluated during echocardiography. The mean CO was 35% decreased in SSI mice as compared to sham-operated mice 24 hours after CLP. The mean CO values from SSI mice treated with either verapamil or dantrolene was considerably improved, with values similar to those observed in sham-operated mice treated with VP and DT at 24 hours after CLP. The mean HR was slightly reduced in SSI mice when compared to sham-operated mice 24 hours after CLP injury and treatment with either verapamil or dantrolene restored HR to those of sham-operated mice. The mean body temperature (°C) of SSI mice was significantly reduced, when compared to sham-operated controls 24 hours after CLP. This difference in body temperature was restored with treatment of either verapamil or dantrolene.

**Table 1:** Major hemodynamic variables in mice 24h after CLP.

	Sham	SSI	SH+VP	SSI+VP	SH+DT	SSI+DT
HR (bpm)	562±36.09	505±47.88	590±16.43	588±22.10	568±61.99	553±32.01
CO (mL/min)	14.57±3.74	9.15±3.78*	16.48±1.08	14.11±2.82	15.08±3.16	13.37±3.73
Body temperature (°C)	36.2±0.36	33.0±2.40*	36.8±0.46	36.4±1.02	36.0±0.80	35.5±1.46

Mice subjected to CLP or sham-operation and untreated or treated with either verapamil (VP) or dantrolene (DT).

Echocardiography procedures were performed in sham-operated or septic mice at the indicated time points as described in materials and methods.

* *P*<0.001 vs. sham.

HR, heart rate; bpm, beats per minute; CO, cardiac output; °C, body temperature.

### Survival rate

**Figure 7:**
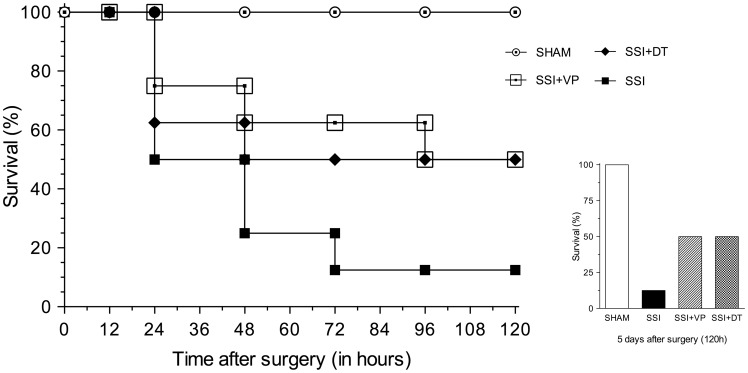
Verapamil and dantrolene improved survival rate in mice submitted to CLP-induced sepsis. Sham-operated untreated mice presented a 100% survival rate throughout the analyzed period of 120 hours. SSI mice had a survival rate of 50% at 24 hours after surgery, decreasing to 10% at 96 hours and remaining steady at this rate until 120 hours after surgery. In contrast, SSI mice treated with verapamil showed a survival rate around 75% at 24 hours, decreasing to 50% at 96 hours and remaining steady at this rate until the end of the experimental period. Similarly, SSI mice treated with dantrolene disclosed a survival rate around 60% at 24 hours, decreasing to 50% at 96 hours and remaining steady at this rate until 120 hours after CLP. The bar graph illustrates the survival rate for each group at 5 days after the surgical procedure. (n = 8 animals per group).

The remarkable protection in all of the physiological parameters usually provided by calcium channel blockers dantrolene and verapamil prompted us to evaluate their effects on the resistance and/or susceptibility of mice subjected to severe sepsis induced by CLP. For these studies, survival rates of both sham-operated and septic mice, treated and untreated, were assessed over a period of 5 days (120 hours) after sham-operation or CLP. Figure 7 shows that SSI mice had a survival rate of 50% at 24 hours after surgery decreasing to 10% at 96 hours; verapamil-treated SSI mice showed a survival rate around 75% at 24 hours decreasing to 50% at 96 hours; and dantrolene-treated SSI mice presented a survival rate around 60% at 24 hours decreasing to 50% at 96 hours after CLP. All sham-operated untreated mice survived throughout the experimental period.

## Discussion

The present study demonstrates for the first time that [Ca^2+^]_i_ in cultured neonatal mice cardiomyocytes markedly increase in response to the addition of serum from septic mice. The acute increase is about 50% and remains elevated by 20% 24 hours later. Higher amplitude of L-type Ca^2+^ channel currents, increased Ca^2+^ release or reduced uptake by the SR or both, may explain an increased [Ca^2+^]_i_ after septic serum addition. At two minutes after septic serum addition to the culture medium, the marked increment of mean [Ca^2+^]_i_ was due to increased diastolic concentration of the ion, the systolic levels being similar to those before septic serum. The diastolic calcium increased, giving rise to the decrease of the amplitude of the calcium transients, probably reflecting decreased calcium uptake by the sarcoplasmic reticulum [27] associated with decreased myocyte shortening [28] at this very beginning of the septic serum challenge imposed to the cultured cardiomyocytes. However, the frequency of Ca^2+^ transients in experimental and control cells was not different. In contrast, at 24 hours after septic serum, there was an effective mean increase of [Ca^2+^]_i_ due to a rise of both systolic and diastolic ion levels. But at this point of the experiment, the amplitude of systolic and diastolic [Ca^2+^]_i_ levels and frequency of Ca^2+^ transients were similar in both experimental and control conditions. A >2-fold increase in [Ca^2+^]_i_ in smooth muscle cells was previously demonstrated in intact perfused rat thoracic aorta excised within the first 24 hours after CLP-induced sepsis as compared to sham controls, which was blocked immediately following the addition of dantrolene to the perfusion medium [17]. Moreover, an i*n vivo* study detected an early almost twofold increase (24 hours after septic process) of calcium concentration, measured with NMR spectroscopy, in brains of adults rats submitted to CLP-induced sepsis in comparison with sham animals [19]. These changes were associated with injury of approximately 0.25% of glial cells. More recently, increased [Ca^2+^]_i_ was disclosed in cultured human adipocytes after LPS stimulation that was partially blocked with verapamil and blocked almost completely with 2-aminoethoxydiphenyl borate, a blocker of store-operated calcium entry and inositol triphosphate-mediated release [16]. Increased Ca^2+^ leakage from the sarcoplasmic reticulum was observed in rat cardiomyocytes isolated in the late phase of CLP-induced sepsis, which was suggested to contribute to decreased myocyte shortening in sepsis [15]. More recently, Ca^2+^ trafficking abnormalities characterized by increased sarcoplasmic reticulum Ca^2+^ release associated with a rise in mitochondrial Ca^2+^ content was demonstrated in cardiomyocytes isolated from rats treated with LPS [18]. It was proposed that sarcoplasmic reticulum Ca^2+^ mishandling is likely to be an early event after intravenous administration of LPS to rats, thereby contributing to mitochondrial Ca^2+^ overload with metabolic failure and consequent cardiac dysfunction.

Indirect evidence of elevated calcium concentration in sepsis has been obtained by several studies showing increased activities of calcium-dependent proteases in tissues of septic animals, such as calpain [29] and glycogen phosphorylase-b kinase [30]. The immunofluorescence findings in the present study demonstrated an increased expression of calpain-1 in cardiomyocytes submitted to septic serum challenge as compared with that observed in cardiomyocytes tested with control serum. These alterations were corroborated by the Western blotting results showing a marked increment in calpain-1 level in cultured cardiomyocytes subjected to septic serum in comparison to those subjected to control serum. Calpains are calcium-activated neutral cysteine proteases with two major isoforms besides tissue specific forms: (a) calpain-1 or µ that requires micromolar Ca^2+^ concentrations for activity and (b) calpain-2 or m that requires millimolar Ca^2+^ concentrations [13]. Binding of Ca^2+^ to calpain induces the release of constraints that are imposed by domain interactions and results in activation. Many of the cytoskeletal proteins involved in linking the cytoskeleton to the plasma membrane as well intermediate filament proteins are cleaved rapidly by calpains. Cultured neonatal mice cardiomyocytes exposed to septic serum displayed decreased immunostaining for dystrophin in comparison with cardiomyocytes exposed to normal serum. This was confirmed by Western blotting that showed substantially decreased dystrophin in cardiomyocytes stimulated with septic serum in comparison with values in cardiomyocytes stimulated with serum from sham-operated mice. Calpains can induce proteolysis of dystrophin very rapidly when appropriately activated [31]. Moreover, the immunofluorescence study showed that cultured cardiomyocytes stimulated with septic serum showed disruption and rearrangement of F-actin filaments causing cell shape changes with bleb formation as an early event. Previously, an elevation in calcium ion concentration *in vitro* was associated with injury in different cell types such as rabbit cardiomyocytes, rabbit proximal tubular cells and rat hepatocytes with formation of blebs and cell death. [32], [33], [34]. Likewise, cultured rat proximal tubule cells showed disruption of F-actin filaments labeled with Alexa Fluor 594 and bleb formation when treated with mercuric chloride (HgCl_2_), a highly toxic and corrosive chemical substance that raises free cytosolic ionized calcium and activates calpain [35].

*In vivo* studies were undertaken to evaluate indirectly the occurrence of elevated calcium myocardial concentration by determining the expression and amounts of calpain-1 in animals submitted to CLP-induced sepsis in comparison with sham-operated controls, either untreated or treated with verapamil or dantrolene. Markedly increased calpain-1 at 6 and 24 hours after CLP were observed in septic mice, confirming the *in vitro* observations. The levels of calpain-1 in septic mice treated with verapamil 6 hours after CLP were similar to the values observed in sham-operated and treated mice. However, treatment with verapamil 24 hours after CLP caused a decrease in the mean amount of calpain-1 compared to the values in sham-operated and sham-operated treated with verapamil or dantrolene. Similarly, treatment with dantrolene decreased cardiac calpain-1 amounts at either at 6 or 24 hours after severe sepsis induced by CLP in comparison with sham-operated and sham-operated treated animals.

The amounts of dystrophin, myosin heavy chain, and sarcomeric actin were evaluated 24 hours after surgery in both sham-operated and septic untreated hearts. The amount of dystrophin was strikingly reduced in septic hearts at 24 hours after sepsis induction as compared with the mean value in hearts from sham-operated mice, corroborating previous data from our laboratory [9], [10]. This decrease is a direct consequence of calcium-dependent calpain-activity that cleaves dystrophin very rapidly when the calcium concentration is compatible with its activation [13], [31]. This interpretation is reinforced by observations in the present study showing that in septic mice treated with verapamil or dantrolene 2 hours after surgery, heart dystrophin levels were similar to those in hearts from sham-operated mice treated with verapamil or dantrolene and sham-operated untreated mice.

Similarly, the contractile myofibrillar proteins, sarcomeric actin, and myosin were markedly reduced 24 hours after CLP, confirming observations from a previous study from our laboratory [9]. Levels of sarcomeric actin and myosin heavy chain in the hearts from septic mice treated with verapamil or dantrolene and in the hearts from sham-operated animals treated with verapamil or dantrolene were not different from the mean amount of the myofibrillar contractile proteins in the hearts of sham-operated untreated mice. Studies *in vitro* have clearly demonstrated that myosin heavy chain is degraded very slowly by calpains and sarcomeric actin is not degraded at all [13]. However, denatured myosin and actin are rapidly degraded indicating that denaturation affects rates of cleavage by calpains. On the other hand, previous investigation support the theory that calpains might be the degradative system responsible for myofibril destabilization by other proteins that are important in the structural integrity of the sarcomere [36], i.e., actin and myosin are poor substrates to calpain but other proteins important for sarcomere integrity, such as titin and nebulin, are excellent calpain substrates [37]. Sepsis has been shown to increase the expression calpain genes, to disrupt Z-bands, and to enhance the release of myofilaments in skeletal muscle [38], which supports a role for calpain activation in the septic process.

In this study, electron microscopy provided morphological evidence of diffuse foci of sarcomere disruption, blurring and breakage of Z-bands, pronounced formation of autophagolysosomes, and increased number of electron-dense granules in the mitochondrial matrix in hearts of untreated septic mice 24 hours after injury. Previous work demonstrated that the disruption of the sarcomere followed by release of actin and myosin plays an important role in sepsis induced skeletal muscle wasting [39]. Actin and myosin from the sarcomere are first disassembled by calpain or caspase and then ubiquitinated and degraded by proteasome [36], [37], [40], [41] The failure or overwhelming of the ubiquitin-proteasome system (UPS) to clear ubiquitinated and aggregated proteins leads to autophagy, which assists UPS, i.e., cytoplasmic contents are aggregated and ubiquitinated proteins are sequestered inside specialized vacuoles called autophagosomes and following this delivered to the lysosome for degradation forming the autophagolysosomes [36], as seen in the present study. Previously, we demonstrated increased perinuclear autofluorescent lipofuscin in cardiomyocytes of human septic hearts [5]. Reactive oxygen species are known to promote lipofuscin pigment formation representing oxidized/cross linked proteins. Similarly, excessive accumulation of autophagosomes has been demonstrated in cardiomyocytes of failing human hearts, which might contribute to autophagic cell death and the development of contractile dysfunction in failing hearts [42]. Moreover, Z-bands, which define the lateral borders of the sarcomeres, are involved in sarcomere organization, force generation and transmission, and act as an interface between contractile proteins, other cytoskeletal proteins, and signaling proteins [43]. A wide variety of Z-band protein mutations are associated with the development of several cardiomyopathies, such as dilated cardiomyopathy, hypertrophic cardiomyopathy, right ventricular cardiomyopathy, and muscular dystrophies [13], [44]. Furthermore, Z-bands might also have an important role in normal protein turnover via the UPS and autophagy. The electron dense granules in mitochondrial matrix correspond to calcium deposits [45]. The increased [Ca^2+^]_i_ in septic heart may lead to mitochondrial calcium accumulation that decreases ATP production and increases mitochondrial generation of reactive oxygen species which further decreases ATP production [46]. This may result in disruption of plasma membrane and cell death. Moreover, increased mitochondrial calcium as occurs in ischemia has been shown to increase autophagy.

Cardiac function in septic and sham-operated mice, untreated or treated with either verapamil or dantrolene, was evaluated through the echocardiographic parameters ejection fraction (FE), a measure of the efficacy of the heart, and fractional shortening (FS), an index of left ventricle contractility. Both EF and FS values in mice submitted to CLP-induced sepsis were similar to those observed in sham-operated animals at 12 hours after CLP surgery. However, reductions in both EF and FS were observed in septic mice in comparison with sham-operated mice at 24 hours after injury. The treatment of septic mice with either verapamil or dantrolene completely abrogated EF and FS changes with mean values similar to those observed in sham-operated untreated and treated animals.

Numerous studies have pointed out the physiopathologic role of dystrophin with its associated glycoproteins on myocardial contractile performance. The lack of dystrophin in the hearts of patients with Duchenne muscular dystrophy (DMD) as well as in *mdx* mice constitutes the structural basis for the development of dilated cardiomyopathy [47], [48], [49], [50]. Also, dystrophin loss has been related to end-stage cardiomyopathies and proposed as a common route for myocardial dysfunction and progression to advanced heart failure [51], [52]. Besides, alteration of contractile proteins might be a major factor determining the development of heart failure, since these alterations can have direct impact on the structural integrity of cardiomyocytes and consequently on reduction of contractile function in the failing heart [53], [54]. The cardiac output (CO), heart rate (HR) and body temperature (°C) assessed during the echocardiographic study all were reduced in septic mice as compared to sham-operated controls at 24 hours after CLP. However, both verapamil and dantrolene treatments restrained CO, HR and BT alterations with mean values similar to those observed in sham-operated untreated and treated mice.

Moreover, the survival rate of septic mice treated with verapamil or dantrolene was >5 fold than septic untreated mice. The partial prevention of the mortality rate in septic mice treated with calcium channel blockers is likely attributable to other causes of death in this pathological process.

In summary, this study offers novel and mechanistic data to clarify subcellular events that occur in the pathogenesis of septic cardiomyopathy and cardiac depression in severe sepsis. Cultured neonatal mouse cardiomyocytes subjected to serum obtained from mice with severe sepsis presented strikingly higher [Ca^2+^]_i_ and calpain-1 levels associated with decreased expression of dystrophin and disruption and derangement of F-actin filaments and cytoplasmic bleb formation. Severe sepsis induced in mice led to increased expression of calpain-1 in cardiomyocytes. Moreover, decreased myocardial dystrophin, sarcomeric actin, and myosin heavy chain were observed in septic hearts associated with depressed cardiac contractile dysfunction and a very low survival rate. Verapamil and dantrolene prevented the increase of calpain-1 levels and preserved dystrophin, actin, and myosin loss/reduction as well cardiac contractile dysfunction associated with strikingly improved survival rate. Actin and myosin from the sarcomere are first disassembled by calpain and then ubiquitinated and degraded by proteasome or sequestered inside specialized vacuoles called autophagosomes, which are delivered to the lysosome for degradation forming autophagolysosomes. These abnormal parameters emerge as therapeutic targets and their modulation may provide beneficial effects on future cardiovascular outcomes and mortality in sepsis. Further studies are needed to shed light on this mechanism, mainly regarding specific calpain inhibitors, which may provide new interventional pathways to prevent sepsis-induced cardiomyopathy and cardiac dysfunction.
